# In Situ Multiphysical Metrology for Photonic Wire Bonding by Two-Photon Polymerization

**DOI:** 10.3390/ma17215297

**Published:** 2024-10-31

**Authors:** Yu Lei, Wentao Sun, Xiaolong Huang, Yan Wang, Jinling Gao, Xiaopei Li, Rulei Xiao, Biwei Deng

**Affiliations:** 1 Yongjiang Laboratory, Ningbo 315202, China; yu-lei@ylab.ac.cn (Y.L.);; 2 Key Laboratory of Intelligent Optical Sensing and Manipulation of the Ministry of Education & National Laboratory of Solid State Microstructures & College of Engineering and Applied Sciences & Institute of Optical Communication Engineering, Nanjing University, Nanjing 210093, China; wentaosun@smail.nju.edu.cn; 3 Fujian Provincial Key Laboratory of Advanced Materials Oriented Chemical Engineering, College of Chemistry and Materials Science, Fujian Normal University, Fuzhou 350007, China; 4 State Key Laboratory of Mechanics and Control for Aerospace Structures, Nanjing University of Aeronautics and Astronautics, Nanjing 210016, China

**Keywords:** two-photon polymerization, photonic wire bonding, in situ SEM, optical loss, mechanical reliability

## Abstract

Femtosecond laser two-photon polymerization (TPP) technology, known for its high precision and its ability to fabricate arbitrary 3D structures, has been widely applied in the production of various micro/nano optical devices, achieving significant advancements, particularly in the field of photonic wire bonding (PWB) for optical interconnects. Currently, research on optimizing both the optical loss and production reliability of polymeric photonic wires is still in its early stages. One of the key challenges is that inadequate metrology methods cannot meet the demand for multiphysical measurements in practical scenarios. This study utilizes novel in situ scanning electron microscopy (SEM) to monitor the working PWBs fabricated by TPP technology at the microscale. Optical and mechanical measurements are made simultaneously to evaluate the production qualities and to study the multiphysical coupling effects of PWBs. The results reveal that photonic wires with larger local curvature radii are more prone to plastic failure, while those with smaller local curvature radii recover elastically. Furthermore, larger cross-sectional dimensions contribute dominantly to the improved mechanical robustness. The optical-loss deterioration of the elastically deformed photonic wire is only temporary, and can be fully recovered when the load is removed. After further optimization based on the results of multiphysical metrology, the PWBs fabricated in this work achieve a minimum insertion loss of 0.6 dB. In this study, the multiphysical analysis of PWBs carried out by in situ SEM metrology offers a novel perspective for optimizing the design and performance of microscale polymeric waveguides, which could potentially promote the mass production reliability of TPP technology in the field of chip-level optical interconnection.

## 1. Introduction

Femtosecond laser-based two-photon polymerization (TPP) is an advanced technique that harnesses the ultra-short pulses and high peak power of femtosecond lasers to achieve 3D lithography in micro/nano scale [1,2,3,4]. Through two-photon absorption, it enables the precise construction of three-dimensional structures in photocurable resins [5,6]. This approach has been successfully employed in the fabrication of various micro/nano optical devices, which has promoted significant advancements in the field of chip-level optical interconnection [7,8,9]. For example, the TPP technology offers a novel solution for creating photonic wire bonding (PWB), enabling interconnections for chip-to-chip or chip-to-fiber scenarios [10,11,12]. Compared to optical coupling methods via precision alignment of microlenses, the advantages of PWB by TPP are significant. It bypasses the laborious mechanical alignment of precision optical elements between photonic chips, thereby improving overall chip yield. Additionally, PWB by TPP provides high production flexibility, making it adaptable to complicated optical configurations in the advanced scenarios of hybrid integration [13]. Most importantly, it facilitates automated large-scale production, paving the way for broader industrial applications.

As the fronts of photonic chip integrations continue to advance, there is an increasing demand for improved PWB performance levels, particularly regarding insertion loss due to reliability issues, which requires further validation by metrology methods. During fabrication, factors such as processing parameters, photoresist shrinkage, and development processes can compromise the dimensional accuracy of PWB microstructures [14,15]. Moreover, the large overhang distances of PWBs weaken the mechanical stability of the structures and might induce unpredictable failures in working conditions. Without advanced metrology methods collecting multiphysical information at the microscale, the optimization of PWBs regarding either optical loss or mechanical reliability is a black-box problem. C. Koos et al. reported that photonic wire bonds are highly stable against mechanical shocks and vibrations, such as those seen in samples dropped from heights of around 1 m [10]. Furthermore, they observed that freestanding waveguide arches with diameters below 2 μm can easily span distances exceeding 100 μm and are unaffected by manual handling with tweezers or intensive rinsing after development. Repeated testing over several weeks with optical powers up to 100 mW showed no degradation in transmission loss [16]. However, previous studies have rarely provided quantitative measurements of the mechanical reliability of PWBs, particularly regarding optical loss under applied deformation. So, existing testing methods are inadequate for real-time observation of deformation and optical loss in PWB under applied stress [16,17]. It is critical to enable the in situ measurement of the optical loss and mechanical reliability of polymer waveguides like PWBs, so that the TPP production can eventually meet the high yield requirements of hybrid integrated photonic chip manufacturing.

With the development of three-dimensional manufacturing technologies at the micro- and nanoscale, in situ scanning electron microscopy (SEM) is becoming an invaluable tool for the measurement and study of new materials and devices [18,19,20]. This method can simultaneously characterize both the mechanical properties and the microstructure of materials, significantly improving upon the “separated” and “static” limitations of traditional testing methods. Recently, in situ SEM testing is evolving toward real-time and dynamic capabilities, allowing researchers to capture the key microstructural changes that dominantly influence the material properties [21,22,23]. This provides deeper insights into material behavior in practical applications. Researchers have utilized in situ SEM to characterize the mechanical properties of TPP-printed microstructures. For instance, R. Cherukuri et al. performed in situ micropillar compression on SU-8 polymer fabricated via TPP, measuring mechanical properties such as modulus, hardness, yield strength, and strain rate sensitivity [24]. Similarly, N. Rohbeck et al. conducted a comprehensive study on the effects of temperature and strain rate on the mechanical properties of micron-sized 3D-printed polymers using Nanoscribe’s negative-tone photoresist IP-Dip [25]. Q. Li et al. employed in situ SEM to measure the remarkable mechanical properties of Cu nanocluster-polymer micro-lattices [26], while Jens Bauer et al. characterized the compressive strength of micro-lattices and shell structures made from alumina–polymer composites [27]. However, there are currently few studies demonstrating the capability to measure both optical and mechanical properties, which is critically needed for the evaluation of PWBs. Specifically, when multiple physical fields are coupled (such as mechanical, thermal, and optical fields) upon microscale devices like PWBs in in situ SEM testing, achieving a comprehensive and precise evaluation remains a significant technical challenge.

In this study, photonic wire bonds were fabricated using two-photon polymerization technology. Through simulation, the taper and bond dimensions were optimized, significantly reducing optical loss, with experimental results showing a minimum loss of 0.6 dB. A novel in situ SEM metrology method is proposed, integrating optical coupling modules and nanomechanical measurements. The dynamic evolution of PWB microstructures is observed under SEM, while their optical and mechanical properties are measured simultaneously. The influences of cross-sectional size and the curvature radius of the photonic wire on the mechanical robustness are examined. Good agreements are achieved with the numerical simulations. The real-time optical losses during deformation were collected to validate the multiphysical coupling in PWBs. The findings revealed that smaller local curvature radii of PWBs are associated with elastic recoveries over vertical indentations of 20 μm, which equals 15% of the photonic wire length. Larger cross-sectional sizes enhanced the load-bearing capacity of photonic wires. With a properly selected local curvature radius and cross-sectional size, a fully recoverable PWB is demonstrated as returning to its initial values, for both geometrical shape and optical loss, after the removal of the force. This study pioneers the use of advanced in situ metrology methods in promoting the precise design and fabrication of TPP printed waveguides. The multiphysical results offer valuable insights for failure analysis and production optimization in advancing high-performance optical interconnect technologies. The combined methodology of microscale manufacturing and in situ metrology establishes a strong foundation for further photonic device optimization.

## 2. Materials and Methods

### 2.1. Materials

The negative-tone photoresist (Vancore B) used for printing the photonic wire bonding (PWB) was purchased from Vanguard Corp. (Vanguard Automation GmbH, Gablonzer, Germany). The photoresist is acylate-based, with a viscosity of 3.0 Pa·s, and the refractive index after curing is 1.5163 at a wavelength of 1550 nm. The single-mode fiber arrays with a mode field diameter of 10.4 μm used to accommodate interconnecting PWBs were purchased from FiberHome Incorporated (Wuhan, China).

### 2.2. PWB Fabrication and Characterization

Two-photon polymerization (TPP) was performed employing a Photonic Professional Sonata1000 (Vanguard) system. Microfabrication of all structures was performed in oil-immersion mode with a femtosecond laser (780 nm, 100 MHZ, 100 fs) focused through a 40× oil objective lens (NA = 1.4; Zeiss, Jena, Germany). Printing was performed with a varied scan speed ranging from 4 to 19 mm s^−1^ and laser power in the range of 2.89 to 5.78 mW. Slicing and hatching were set to 100 nm for all micro geometry. When generating a layer, the spacing between adjacent scanning lines is called hatch distance. The distance between each layer is referred to as the slice distance. Fabricated structures on fiber array substrates were developed for 10 min in PGMEA; this was followed by rinsing with IPA for 5 min. The single-mode fiber core is made of silica (SiO_2_) doped with germanium dioxide (GeO_2_), while the cladding is made of silica (SiO_2_). The mode field diameter is 10.4 ± 0.5 μm at a working wavelength of 1550 nm; the cladding diameter is 125 ± 0.7 μm. Each fiber array contains 16 parallel channels, with a spacing of 250 μm between adjacent fibers and 280 μm between the two arrays. In addition, the fiber end face was designed as a vertical surface and polished. A smooth fiber end face not only reduces reflection loss but also minimizes the likelihood of minor and irregular defects, thereby reducing scattering loss. Using TPP technology, PWBs were printed between the centers of the fiber cores in the FA–FA array. Specifically, a confocal scan was performed over a range of several tens of micrometers near the fiber core, primarily by adjusting the laser’s longitudinal height to determine the core’s position. The polished fiber end face not only facilitates accurate identification of the fiber core during processing but also reduces coupling loss between the fiber and the PWB. The viscosity of the photoresist was measured using a viscometer (MCR302e, Anton Paar, Graz, Austria), and the viscosity samples were kept at a constant temperature of 25 °C for 1 h before testing. The refractive index of the polymer films was measured using an ellipsometer (RC2, J.A. Woollam, Lincoln, NE, USA) over a wavelength range of 210–1690 nm. The morphology of PWB was observed using a Gemini SEM 360 scanning electron microscope (SEM, Zeiss). Before imaging, the structures were sputter-coated with a layer of Pt. approximately 10 nm thick. The mechanical properties of the PWBs were tested via the in situ quantitative PicoFemto (NI-100) (ZEPTOOLS, Tongling, Anhui, China) equipped with a 100 μm customized tungsten carbide cylindrical indenter with a speed of 200 nm s^−1^ inside the SEM. The system has a maximum closed-loop indentation displacement of 100 μm and a maximum load of 100 mN. The three-axis stage carrying the sample has a movement range of up to 10 mm. During the optical-loss testing, a single-frequency light source with a wavelength of 1550 nm (ZG-T-15-100-SM, Hunyuan Technology Co., Ltd., Yuxi, Yunnan, China) was applied to one side of the fiber array, while a storage-type optical fiber power meter (MT-7603-C, Pro’sKit, Shanghai, China) was connected to the other end. The inherent loss of each channel was specified prior to shipping the FA samples. To accurately measure the actual optical loss of the PWB, the loss from the fiber array must be subtracted.

### 2.3. FEM Simulation

The structure coupled to the fiber port is an inverted cone with a circular cross-section. This study calculates the transmission loss of the inverted cone using FDTD (Figure S1a) and creates a circular cylinder with a circular cross-section in the FDE solver (Figure S1b) to evaluate the mode match between the PWB and the fiber spot. The boundary condition is set to “Metal” (as PML boundaries can introduce unphysical modes or small gains/losses, the official guidance suggests choosing the most numerically efficient boundary condition instead of the physically most accurate one). The model has a refractive index of 1.5163, the background refractive index is 1, the mesh size is 0.02 μm, the working wavelength is 1550 nm, and the fiber dimensions and refractive index were based on FiberHome@SMF-G657A1 optical fiber specifications. The cylindrical model’s diameter is scanned over the range shown in Figure S1b, and with a height of 10 μm. The mechanical reliability simulation of PWB was performed using Abaqus software (6.13). The static analysis was used to simulate the compression process due to the low loading speed. The contact algorithm was configured as “general contact”, allowing all surfaces to interact realistically. Additionally, the tangential friction coefficient was set to 0.1, and the expected contact behavior was defined as “hard contact” to better reflect actual conditions. The photoresist material has a Poisson’s ratio of *v* = 0.49, with a Young’s modulus of 2.6 GPa and a yield stress of 0.07 GPa. The eight-node linear brick element (C3D8R) is used for linear-elastic analysis.

## 3. Results and Discussion

### 3.1. In Situ Multiphysical Metrology

**Figure 1:**
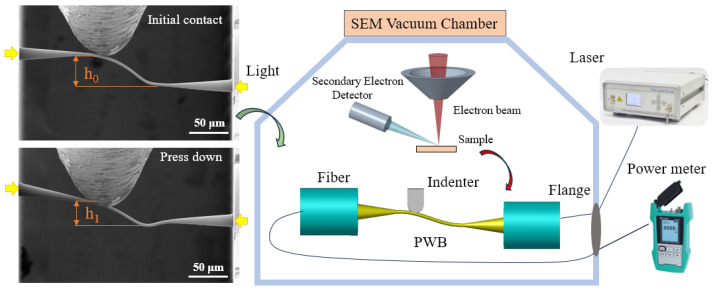
Measurement of optical loss and the mechanical properties of PWBs using in situ SEM.

A novel set of metrology methods for PWBs was designed based on the existing in situ SEM nanomechanical measurement platform, allowing simultaneous capturing of micro/nano scale morphological changes during mechanical loading and optical-loss monitoring. The nanomechanical measurement enables the quantitative evaluation of the mechanical reliability of the PWB, while the simultaneous optical-loss monitoring establishes the correspondence between mechanical perturbation and waveguide performance. The schematic diagram and SEM snapshots of the measurement process are shown in Figure 1. The equipment setup diagram is shown in Figure S2. The two fiber arrays (FAs) with printed interconnecting PWBs are placed on a three-axis piezo stage of the nanomechanical tester beneath a specially shaped indenter with a force sensor. The indenter, made from tungsten carbide, was designed with a cylindrical cross-section for better alignment of the PWB and was milled using a focused ion beam (FIB) SEM to avoid disruptive loading on the curved photonic wires (Figure S3). As the optical measurement module, FA–FA samples connected to PWB were equipped with FC/APC pigtails on both sides. These pigtails were connected to optical fibers outside the SEM chamber through the flange on the chamber wall. One fiber was connected to an external laser with a wavelength of 1550 nm, and the other fiber was connected to a laser power meter. The total optical loss was then calculated as the ratio of the reading of the power meter over the input power from the laser. In a typical mechanical test with optical-loss monitoring, the indenter is driven by displacement control and applies contact pressure to the highest location of the photonic wire. At the same time, SEM imaging is conducted at video rate sufficient for a clear observation of deformation, while the laser power meter reading is collected for optical-loss calculation under varying mechanical loads. As shown clearly in Figure 1, h_0_ is greater than h_1_, indicating that the PWB undergoes deformation. To ensure a reliable connection between the PWB and the optical fiber, the taper size of the PWB near the optical fiber is typically printed in a larger size. In this case, the fiber mode field is 10.4 μm, while the taper size is 14 μm. This design choice serves two purposes: first, to improve mode matching between the fiber and the PWB, and to reduce coupling loss and increase the processing tolerance between them, and second, to increase the contact area between the PWB and the optical fiber, thereby preventing detachment. Additionally, the optimal taper length for this experiment is 75 μm. To enhance the stability of the connection, the taper length is generally designed to be longer, with the extended portion being printed into the optical fiber. The FA–FA distance fixed at 280 μm, a taper length of 75 μm, a bond overhang of 130 μm, and a diameter of 3 μm are shown in Figure 1. Overall, this in situ SEM measurement method captures real-time microscale deformations of working polymeric waveguides, such as PWBs, and correlates stress–strain responses with optical-loss data, offering comprehensive and detailed insights into the multiphysical behavior of PWBs.

### 3.2. Laser Parameters of PWB by TPP

**Figure 2:**
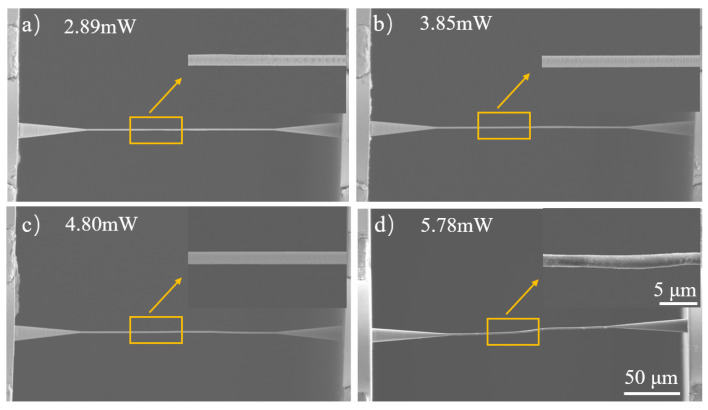
SEM images of PWBs at increasing TPP-process laser powers; the inserted figure is a magnified view.

The femtosecond laser exposure in the process of TPP is crucial to obtaining prints with good surface qualities. The high surface qualities of polymeric waveguides like PWBs then guarantee the desirable optical transmission functions. Therefore, the optimized laser exposure parameters during the TPP process are determined through surface quality examinations of PWBs based on SEM observations. In principle, reduced laser exposure leads to a lower extent of crosslinking of resins and polymeric network formation, which results in either incomplete curing of the intended structure or no curing at all, as shown in Figure S4. As the laser exposure increases, the crosslinking extent also increases, although the accumulation of heat could jeopardize the printing by causing shape distortions or bubble formations. As shown in Figure 2a,b, at relatively low laser powers, the surfaces of the photonic wires are filled with nanometer-level steps, which is a typical sign of poor-quality TPP printing. When the laser power is elevated to 4.80 mW, freestanding photonic wires with smooth surfaces are fabricated (Figure 2c). As the laser power is further increased to 5.78 mW, overexposure is clearly observed, with the cylindrical photonic wire becoming irregularly thickened (Figure 2d). Similarly, the effect of laser scanning speed on the printed quality of PWB was also explored and optimized, as shown in Figure S5. The appropriate printing process is essential for the surface morphology of photonic wire bonding, as shown in Figure S6. As a result, for the TPP experiment in this work, a printing speed of 16 mm/s and a laser power of 4.80 mW were used to fabricate PWBs with uniform diameters and excellent surface quality.

### 3.3. Geometrical Design for Low-Loss PWB

**Figure 3:**
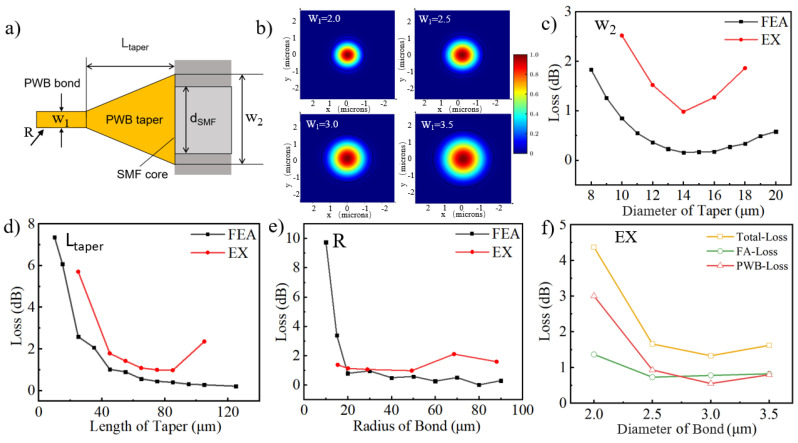
Shows the optical-loss measurements of the PWB. (**a**) provides a schematic of the photonic wire bond design parameters, highlighting the significant difference between the mode field diameters of a standard single-mode fiber (FiberHome@SMF-G657A1) and the PWB. The typical mode field diameter of an SMF is 10.4 μm, while that of the PWB is less than 3 μm. Simulations and experiments were conducted to evaluate the optical loss for varying bond cross-sectional diameters (w_1_) (**b**), taper widths (w_2_) (**c**), taper lengths (L_taper_) (**d**), and bond curvature radii (R) (**e**). (**f**) presents the measured optical losses of printed PWB structures with varying bond cross-sectional diameters in an FA–FA array with a total length of 280 μm, while maintaining the fixed parameters of w_2_ = 14 μm, L_taper_ = 75 μm, and a curvature radius of R = 40 μm.

The optical loss of the PWB directly indicates its optical transmission efficiency. To minimize interconnection losses, the two ends of the PWB must be precisely spatially aligned with the connected photonic components. Firstly, the PWB’s cross-section should match the optical mode diameter of the interconnecting components to ensure optimal photonic signal transmission. Then, the PWB’s trajectory should be carefully planned to avoid optical losses due to sharp bends. These two factors represent the biggest challenge in the design of the PWB. In our work, to ensure mode matching with the optical fiber port, the ends of the PWB are designed with a tapered structure to facilitate efficient optical signal transmission. In a taper with a circular cross-section, the diameter decreases linearly from w_2_ to w_1_ over the taper length (L_taper_), with the tail end matching the diameter of the bond, both being w_1_, as shown in Figure 3a. The bond size determines the light propagation mode in the PWB. For small waveguide diameters, a single mode dominates, characterized by a simpler structure, with the electromagnetic field concentrated at the waveguide’s center. As the diameter increases, higher-order modes begin to appear, leading to increased optical losses. Figure 3b illustrates simulations of the electric field modes for circular waveguides with varying cross-sectional diameters using the FDE method (n_Vancore B_ = 1.5163 at a wavelength of 1550 nm). Enlarging the bond diameter alters the optical field’s mode distribution, reducing the normalized field intensity at the waveguide interface and diminishing interaction with the sidewall, which decreases transmission losses [28]. By optimizing the waveguide diameter, low-loss optical transmission can be achieved within a specific frequency range, thereby enhancing waveguide performance. FDE simulations were also used to calculate the overlap integral between the PWB mode and the single-mode fiber, estimating the optical loss for tapers with different widths (w_2_). As shown in Figure 3c, as the w_2_ size increases, the optical loss decreases to a certain point and then increases. When the fiber diameter reaches 14 μm, the loss reaches its minimum. Additionally, the insertion loss at the interface between the PWB and fiber for different taper lengths was simulated, as shown in Figure 3d. A circular taper cross-section was used, and the insertion loss was optimized by adjusting the taper length. The circular taper cross-section was chosen because it provides better optical transmission efficiency and lower insertion loss. Simulations using the FDTD (Finite Difference Time Domain) method were conducted for different taper lengths, with fixed values of w_1_ = 3 μm and w_2_ = 14 μm, showing that the loss stabilized when the length reached approximately 75 μm. Optical losses for different PWB curvature radii were also simulated and analyzed. As shown in Figure 3e, when w_1_ is fixed at 3 μm, smaller waveguide curvature radii lead to higher optical loss. This is because smaller curvature radii cause more bending and scattering of light within the waveguide, increasing transmission loss. The waveguide curvature radius refers to the minimum bending radius of the bond. As the curvature radii increase, optical loss gradually decreases. When the waveguide curvature radii reach approximately 30 μm, the loss stabilizes, and further increases in curvature radius have little effect. This indicates that once the waveguide curvature radius exceeds a certain threshold, the light’s transmission path becomes smoother, and loss is primarily determined by material absorption and scattering. Overall, in the PWB design, both taper length and bond curvature radii can heavily impact the optical loss. Experiments also show how transmission loss varies with the taper diameter, taper length, and the minimum bending radius of the bond. The overall trend of the losses is consistent with the simulations in Figure 3c–e. The surface roughness of the PWB significantly affects optical loss [29] (see Supporting Information). Additionally, testing the roughness of 3D suspended waveguides is challenging (see Figure S7), and therefore it is not extensively discussed here. In this work, with the FA–FA distance fixed at 280 μm, a taper length of 75 μm and a bond overhang of 130 μm are selected as the optimized design. The local curvature radii of the bond are restricted to 30 μm or larger. After optimization, the PWB was successfully printed, and its optical-loss testing results are shown in Figure 3f. Since both sides of the tail fibers in the FA–FA array printed on the PWB samples are FC/APC ports, they are connected to the laser and the power meter, respectively. The measured loss in this setup is referred to as the total loss. Additionally, each FA sample comes with manufacturer-provided specifications for intrinsic losses per channel, referred to as FA Loss. We define the transmission loss of the PWB as PWB Loss = Total Loss − FA Loss. The experiment measured the total optical loss of the fiber array and the PWB, revealing that the insertion loss of the PWB remained below 3 dB, with a minimum of 0.6 dB at 1550 nm. We also add loss tests in the C-band, which show that the PWB exhibits uniformly low loss in this band (see Figure S8). The results demonstrate that optimized design and precise printing significantly reduce coupling loss, thereby improving the overall optical transmission performance of the system.

### 3.4. Mechanical Resilience and Optical Recovery of PWB

The mechanical resilience of PWBs is a crucial but often neglected evaluation in the reliable TPP production of high-precision chip-level photonic interconnects. In a typical PWB fabrication process, TPP is followed by processes such as development in solvents, multiple rinsing procedures, and cladding by low-refractive-index resins, processes which are not as precisely controllable as the high-resolution TPP printing. To ensure that PWBs maintain their structural integrity through these additional processes, a certain level of mechanical resilience is apparently needed. For example, isopropyl alcohol (IPA) is a solvent regularly used in the rinsing process of TPP, and it has a surface tension of 22 mN/m [30]. For a PWB across a distance of 280 μm, the maximum capillary force suffered by the PWB structure during the drying of IPA would be around 12 μN. In other words, it should be ensured that the PWB structure can elastically recover from such external loads, so that the optimized optical transmission can be retained in the end.

**Figure 4:**
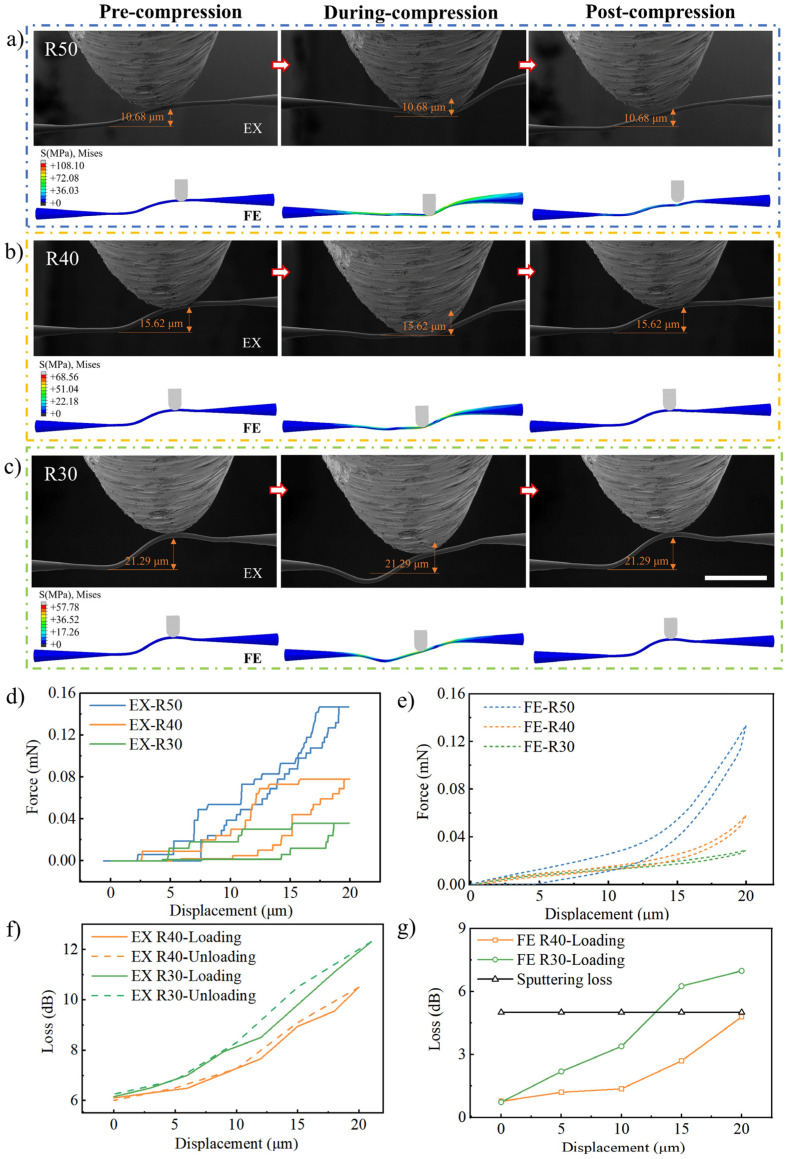
Compression experiments on PWBs with different curvature radii. (**a**) SEM images and finite-element simulations of the PWB during compression with a curvature radius of 50 μm (**a**), 40 μm (**b**), and 30 μm (**c**). (**d**,**e**) Force–displacement curves of the PWB under experimental (EX) and finite-element (FE) simulation conditions. (**f**,**g**) Optical loss of the PWB during the compression process under experimental and simulation conditions. The scale bars: 40 μm.

Therefore, the impact of the geometrical parameters of PWBs on mechanical resilience is examined with in situ SEM measurements. PWBs with different curvature radii were tested to observe their deformation under specific compression. When the curvature radius was 50 μm, the PWB failed to recover after a 20 μm compression, exhibiting plastic deformation, as shown in Figure 4a. In contrast, with radii of 40 μm and 30 μm, the PWBs fully recovered, displaying elastic deformation, as shown in Figure 4b,c.

Due to the inherent elastic properties of photoresist material, PWB structures with smaller curvature radii exhibit longer overall elastic deformation lengths, resulting in reduced total strain under the same applied displacement and minimal plastic deformation. Once the applied force is fully withdrawn, no residual plastic deformation remains. In contrast, PWBs with larger curvature radii experience increased total strain and are more likely to exceed the elastic strain limit, transitioning into plastic deformation. Even after the pressure is released, residual plastic deformation persists, predominantly localized at the indenter’s point of contact. Both simulations and experimental results confirm this behavior. Photonic wire bonding (PWB) with different curvature radii was tested to observe the samples’ deformation under a compression distance of 40 μm or even greater, and the experiments indicated that all exhibited plastic deformation, as shown in Figures S9 and S10. These results indicate that PWBs with larger curvature radii have lower elastic displacement capacity in the vertical direction and are more prone to permanent deformation, and vice versa. Figure 4d and Figure 4e show the experimental and numerically simulated force–displacement curves for PWBs with different curvatures, respectively. The resemblance of compression and recovery behaviors indicates a mutual validation between experimental and simulated data. The in situ multiphysical measurement used in this work allows simultaneous monitoring of optical loss during the compression and recovery of PWB. As seen in Figure 4f, during compression, optical loss increases proportionally with the indentation displacement. Once the indenter is retracted, causing the PWB structure to recover to its initial shape, the optical loss also returns to its initial value. In Figure 4g, a platinum coating was applied to the PWB to enhance the visibility of its shape evolution under compression in the SEM. This coating introduced an additional optical loss of approximately 5 dB. When this loss was factored into the analysis, the simulation results closely matched the experimental data, confirming the model’s accuracy. The simulation process of the optical loss of PWBs with varying curvatures under compression is shown in Figure S11. However, when the curvature radius was 50 μm, the initial loss was 5.64 dB. Upon pressing by 20 μm, plastic deformation occurred, resulting in an irreversible optical loss of 6.97 dB after the indenter was withdrawn. This is direct evidence that the elastic recovery of the PWB can guarantee preserved optical transmission with no accumulated effects as to the losses. It is worth noting that the platinum coating for SEM observation, despite causing a higher initial optical loss, does not affect the full-optical-recovery determination when mechanically fatigued along with the PWB.

**Figure 5:**
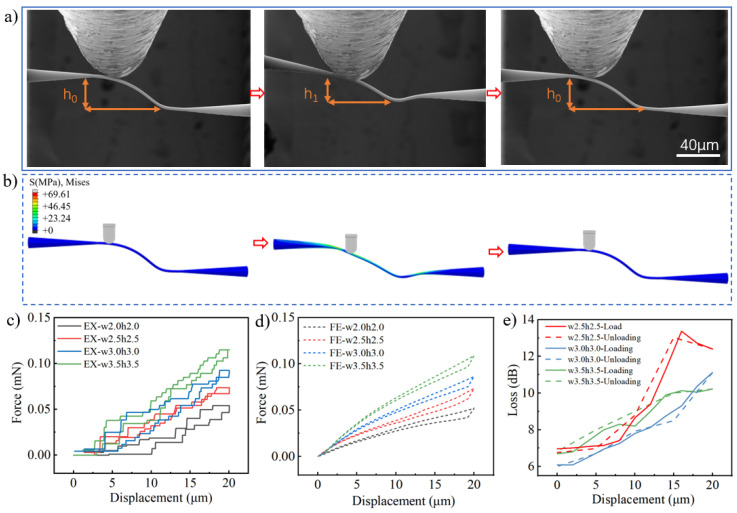
Compression experiments on PWBs with different bond diameters. (**a**) This SEM image shows the deformation process of PWB during the compression process; the PWB with a curvature radius of 30 μm exhibits reversible deformation when compressed by 20 μm. (**b**) Finite-element simulations of the compression response of the PWB structure in Figure (**a**); (**c**,**d**) The force–displacement curve of PWB of under experimental and simulation conditions; (**e**) The optical loss corresponding to PWB during the compression process. The scale bars: 40 μm.

To further explore the variation in PWB mechanical resilience with changes in bond cross-sectional size, the cross-sectional size of the bond was altered while keeping all other PWB dimensions constant. Figure 5a shows the SEM images of the PWB with a bond diameter of 3 μm before and after a 20 μm compression. It can clearly be observed that the PWB undergoes significant deformation during the compression process, but fully recovers once the indenter is removed, indicating that elastic deformation has occurred. Figure 5b presents the simulation results of the PWB during compression, in which stress is primarily concentrated on the upper and lower surfaces of the bond and at the junction with the taper. Figure 5c,d shows the force–displacement curves obtained from the experiments and simulations, respectively, which are in good agreement. Additionally, the compressive strength of the PWB increases as the bond size increases. During the compression of PWB, its optical loss was also measured, as shown in Figure 5e. As the compression increased, the deformation of the PWB led to a corresponding rise in optical loss. However, once the indenter was lifted and the PWB recovered its original shape, the optical loss was able to return to its initial value.

## 4. Conclusions

Here, in order to achieve the goal of measuring the multiphysical responses of photonic wire bonding (PWB) fabricated by two-photon polymerization (TPP), a novel in situ SEM technique, integrating optical and nanomechanical measurements, is developed. With the help of real-time observations of PWB morphological changes, concurrent analysis is conducted on the relationship between the force–displacement curve and optical loss. The optimal printing process is identified. The optical loss of the PWB is ultimately reduced to a value as low as 0.6 dB. The mechanical resilience and optical recovery of the PWB are also investigated. It is observed that when the PWB curvature radius is reduced to below 40 μm, the photonic wire can fully recover, avoiding undesired plastic deformation. Further analysis reveals that the mechanical resilience of the PWB increases with bond cross-sectional size (from 2 to 3.5 μm), which guarantees the necessary load-bearing capacity. Within the elastic deformation range, the optical loss of the PWB increases under loading but returns to its initial value once the load is retracted. These detailed studies on the relationship between the mechanical reliability and optical transmission of PWBs facilitate the optimization of design and process parameters, leading to new industrial prospects in which advanced metrology methods boost the knowledge, and eventually the yield, of micro/nano scale chip-level manufacturing.

## Data Availability

The original contributions presented in the study are included in the article/Supplementary Materials, further inquiries can be directed to the corresponding authors.

## Supplementary Materials

The following supporting information can be downloaded at: https://www.mdpi.com/article/10.3390/ma17215297/s1, Figure S1: Transmission loss model of the inverted cone based on FDTD; Figure S2: The equipment diagram of in situ scanning electron microscopy; Figure S3: SEM image of in situ indenter; Figure S4: SEM Image of failed print of photonic wire bonds; Figure S5: SEM images of PWB at increasing laser speed; Figure S6: Transmission loss varies with the taper diameter, taper length, and the minimum bending radius of the bond; Figure S7: SEM images of a smooth bond and rough bond; Figure S8: The experiment measured the optical loss of the PWB in the C-band; Figure S9: Compression experiments on PWBs with different curvature radii; Figure S10: SEM images and force–displacement curves of the PWB under experimental conditions; Figure S11: The simulation process of the optical loss of photonic wire bonding with varying curvatures.
