# Supplementary material for: In Situ Multiphysical Metrology for Photonic Wire Bonding by Two-Photon Polymerization
Source: Materials (Basel). 2024 Oct 31;17(21):5297. doi: 10.3390/ma17215297 (PMC11547578; doi:10.3390/ma17215297)
Supplement: Supplementary file 1 [file materials-17-05297-s001.zip › materials-3242513-supplementary.pdf]

materials

Supporting information

In-Situ Multiphysical Metrology for Photonic Wire Bonding by Two Photon Polymerization

Yu Lei [1,†], Wentao Sun [2,†], Xiaolong Huang [3], Yan Wang [1], Jinling Gao [4], Xiaopei Li [3,*], Rulei Xiao [2,*] and Biwei Deng [1,*]

[1] Yongjiang Laboratory, Ningbo 315202, China; yu-lei@ylab.ac.cn (Y.L.)
[2] Key Laboratory of Intelligent Optical Sensing and Manipulation of the Ministry of Education & National Laboratory of Solid State Microstructures & College of Engineering and Applied Sciences & Institute of Optical Communication Engineering, Nanjing University, Nanjing 210093, China; wentaosun@smail.nju.edu.cn
[3] Fujian Provincial Key Laboratory of Advanced Materials Oriented Chemical Engineering, College of Chemistry and Materials Science, Fujian Normal University, Fuzhou 350007, China
[4] State Key Laboratory of Mechanics and Control for Aerospace Structures, Nanjing University of Aeronautics and Astronautics, Nanjing 210016, China
* Correspondence: lixiaopei@fjnu.edu.cn (X.L.); xrl@nju.edu.cn (R.X.); biwei-deng@ylab.ac.cn (B.D.)
† These authors contributed equally to this work.

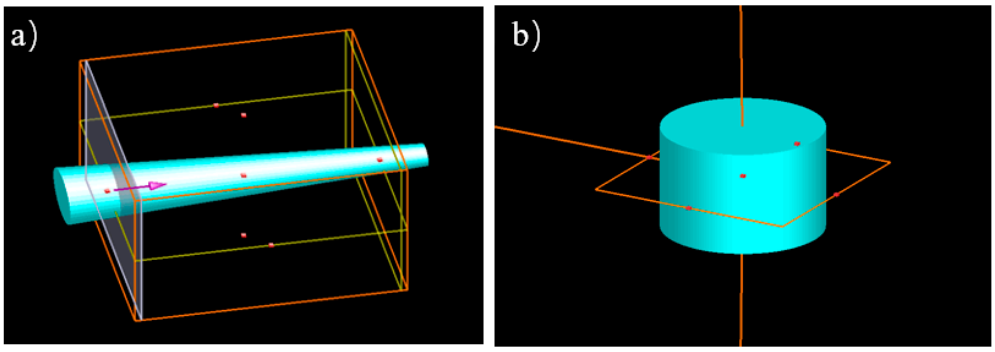

Figure S1 Transmission loss model of the inverted cone based on FDTD (Figure 1(a)). Model of a circular cylinder with a circular cross-section in the FDE solver (Figure 1(b)).

In-situ SEM measurement system consists of a nanomechanical module and an optical coupling module, as illustrated in the equipment diagram (Figure S2). The fiber arrays printed with PWBs are placed on the sample stage of the nanomechanical stage, and their position is adjusted using a three-axis piezo stage. The indenter is mounted on a force sensing module, with its displacement controlled electrically. FA-FA samples connected to PWB are equipped with FC/APC pigtails on both sides. These pigtails are connected to optical fibers outside the SEM chamber through the flange on the chamber wall. One fiber is connected to an external laser of the wavelength of 1550 nm; and the other fiber is connected to a laser power meter.

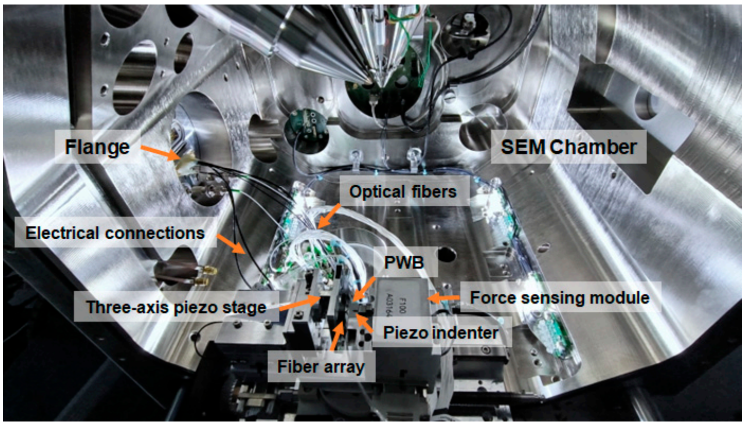

Figure S2 The equipment diagram of in-situ scanning electron microscopy.

In this experiment, the indenter was made from tungsten carbide and designed with a cylindrical cross-section for better alignment of the PWB, as shown in Figure S3. The diameter of the cylindrical cross-section is 100 μm. This design offers two main advantages. First, the large-size indenter makes it easier to align the suspended photonic wires, reducing operational difficulty and minimizing errors. Second, the cylindrical cross-section provides a larger contact area, which helps distribute the applied pressure more evenly, thus preventing uneven stress concentrations on the photonic wires and reducing the risk of damage. This precise design and implementation ensure more accurate and reliable alignment and positioning of the photonic wires during the experiment, enhancing the repeatability and credibility of the results.

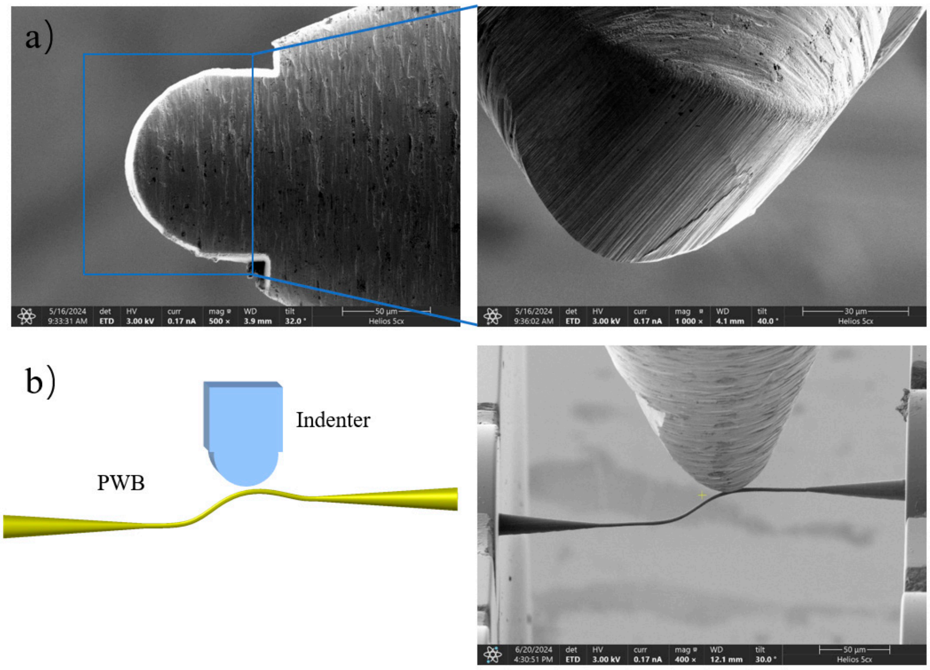

Figure S3. SEM image of in-situ indenter. (a) SEM images of the in-situ indenter and the local morphology, (b) Schematic and SEM measurements of the mechanical properties of the PWB.

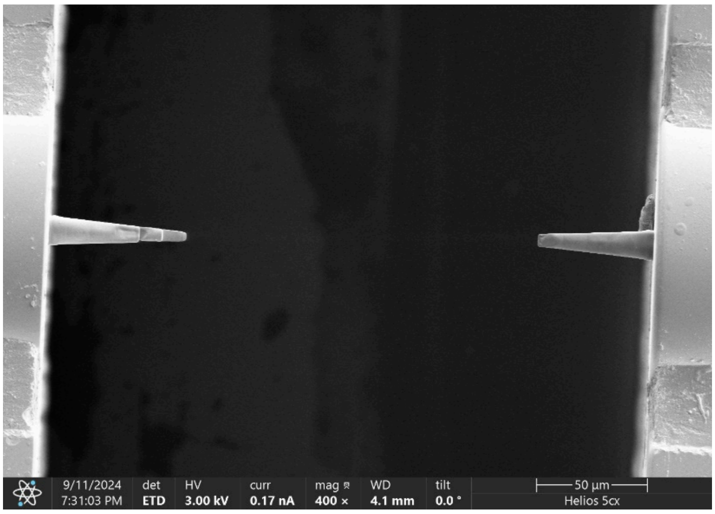

Figure S4. SEM Image of Failed Print of Photonic Wire Bonds.

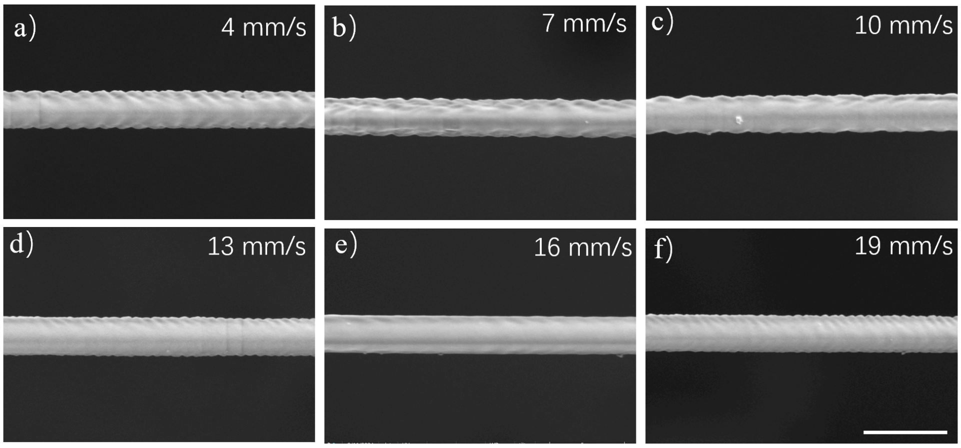

Figure S5. SEM images of PWB at increasing laser speed.

Experiments showing how transmission loss varies with the taper diameter, taper length, and the minimum bending radius of the bond in in Figure 3(c), (d), and (e). The overall trend of the losses is consistent with the simulations. Notably, when the taper length increases to 105 μm, the loss increases rather than decreases. This may be due to the added length causing the taper to sag under gravity, leading to misalignment with the fiber in the Z-direction, resulting in additional loss. Furthermore, when the bending radius increases beyond 68 μm, the transmission loss also increases. In the experiment, to maintain consistency in taper-related variables, the bond shape was adjusted from "arch-like" to "flat-like" to achieve a larger bending radius. Although the curvature increased, the inherent layered printing process faced greater slicing lengths, making polymer drift more likely during fabrication. This caused deviations from the intended PWB model, leading to additional transmission losses. In practical applications, such large bending radii are unlikely to occur; this test was performed merely to verify the simulation results.

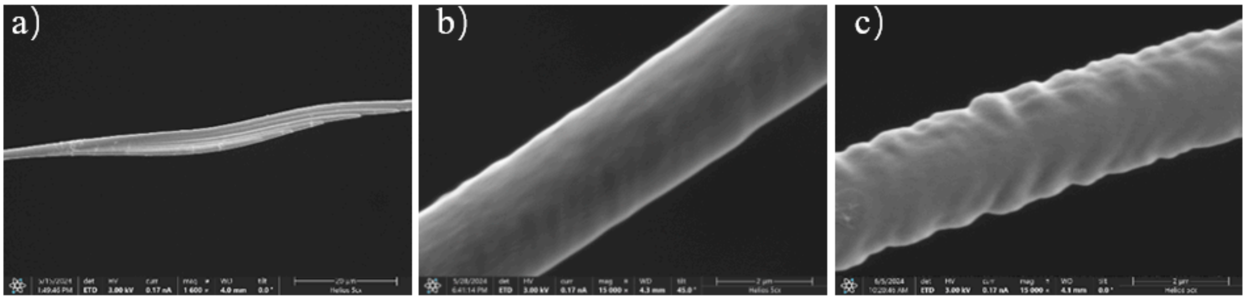

Figure S6. Transmission loss varies with the taper diameter (a), taper length (b), and the minimum bending radius of the bond (c).

The surface roughness of PWB indeed significantly affects the optical loss. Yurii A. Vlasov et al. also studied the effect of surface roughness on transmission loss of straight waveguides [28]. By referencing the models of Marcuse, Payne, and Lacey, they proposed that a perfect dielectric waveguide only supports guided modes without mode conversion. Any defects, such as local variations in refractive index, deviations of the waveguide boundary from a straight line, or interface defects due to minor changes in refractive index, can couple the guided mode with other non-guided modes, transferring energy from the desired guided mode to undesired guided modes and radiation modes, thereby increasing loss. The relationship between surface roughness (σ) and transmission loss (α) can be evaluated: $\alpha = \frac{4\sigma^2 h^2}{\beta(r + 2/p)}$ where σ is the interface roughness, β is the modal propagation constant, and r is the waveguide thickness, while h and p are the transverse propagation constants in the core and cladding, respectively. For a bent waveguide with a fixed curvature, the impact of additional radiation loss can be evaluated by the following equation: $\alpha = K \cdot \exp(-cR)$, where $c = \beta(2\Delta n_{eff} / n_{eff})$ where K depends on the refractive indices of the cladding and core, and on the waveguide thickness, while β is the modal propagation constant. Δn_{eff} is the difference between the modal effective index n_{eff} and the cladding index. To avoid optical loss from abrupt turns or bends, the trajectory of the PWB waveguide is 3D and typically features gradually changing curvature. While the theory for planar waveguide bending is well established, there is no available theory for waveguides bent in three dimensions [29]. However, a qualitative analysis suggests that lower surface roughness results in reduced waveguide transmission loss, though quantitative calculations remain complex. Additionally, testing the roughness of 3D suspended waveguides is challenging, and therefore this is not extensively discussed in this work. We utilized ImageJ software to analyze the SEM images of the printed photonic wire bonds, measuring the line edge roughness. This enabled us to evaluate the surface smoothness of the printed PWB within a defined range.

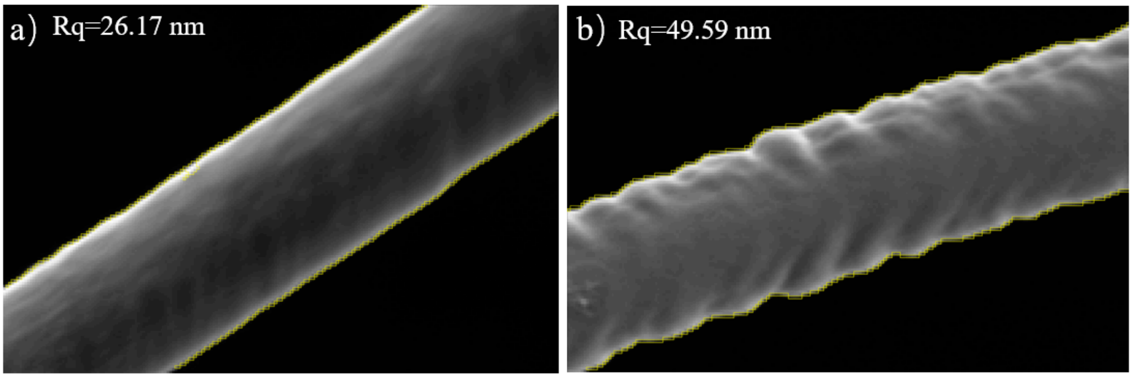

Figure S7. SEM images of a smooth bond (a) and rough bond (b).

Notably, when the taper length increases to 105 μm, the loss increases rather than decreases. This may be due to the added length causing the taper to sag under gravity, leading to misalignment with the fiber in the Z-direction, resulting in additional loss. Furthermore, when the bending radius increases beyond 68 μm, the transmission loss also increases. In the experiment, to maintain consistency in taper-related variables, the bond shape was adjusted from "arch-like" to "flat-like" to achieve a larger bending radius. Although the curvature increased, the inherent layered printing process faced greater slicing lengths, making polymer drift more likely during fabrication. This caused deviations from the intended PWB model, leading to additional transmission losses. In practical applications, such large bending radii are unlikely to occur; this test was performed merely to verify the simulation results.

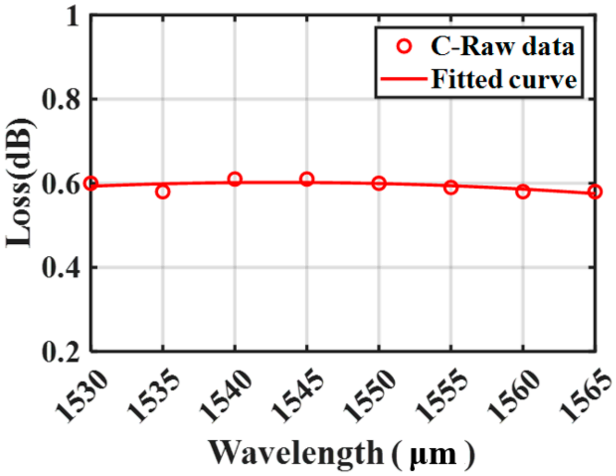

Figure S8. The experiment measured the optical loss of the PWB in the C-band.

PWBs with different curvature radii were tested to observe their deformation under a compression distance of 40 μm. It was found that the photonic wire bonding with a curvature radius of 30 μm exhibited slight deformation upon pressure release, indicating that PWB of different curvatures (30, 40, and 50 μm) all underwent plastic deformation without recovery, as shown in Figure S9 (a). The force-displacement curves were consistent across the experiments presented in Figure S9 (b). Under significant compression distances (40 μm), the PWB experienced plastic deformation, resulting in irreversible optical loss, as shown in Figure S9 (c). The PWB with a curvature radius of 30 μm after breaking following a compression of 48 μm as shown in Figure S10. At this point, the optical loss is significant, measuring -54.39 dB, making it unsuitable for optical transmission.

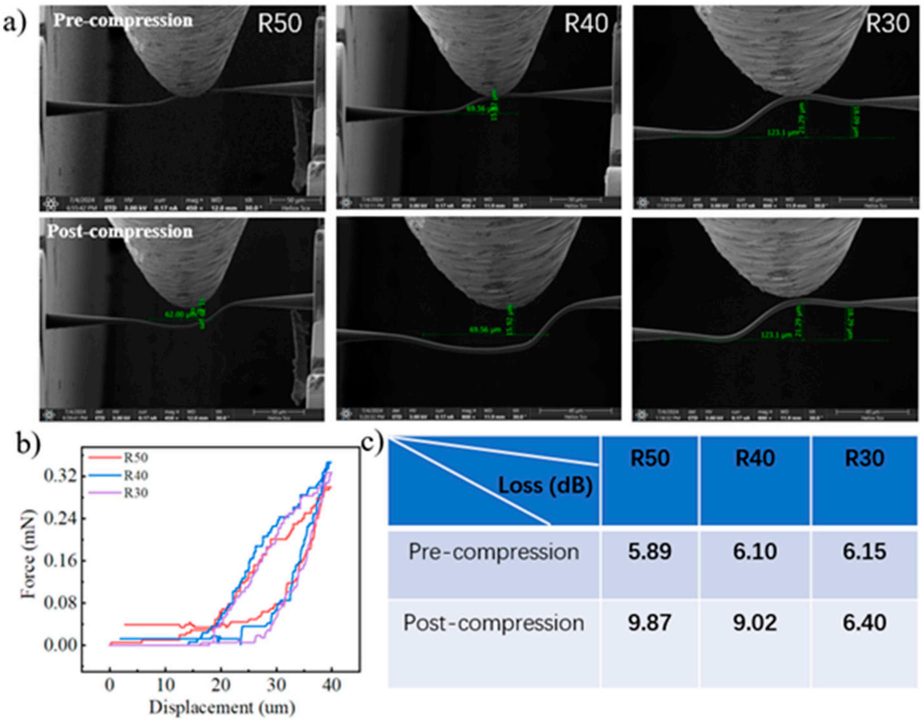

Loss (dB)	R50	R40	R30
Pre-compression	5.89	6.10	6.15
Post-compression	9.87	9.02	6.40

Figure S9. Compression experiments on PWBs with different curvature radii. (a) SEM images of the PWB during compression with a curvature radius of 50 μm, 40 μm, and 30 μm. (b) Force-displacement curves of the PWB under experimental conditions. (f) Optical loss corresponds to the PWB during the compression process.

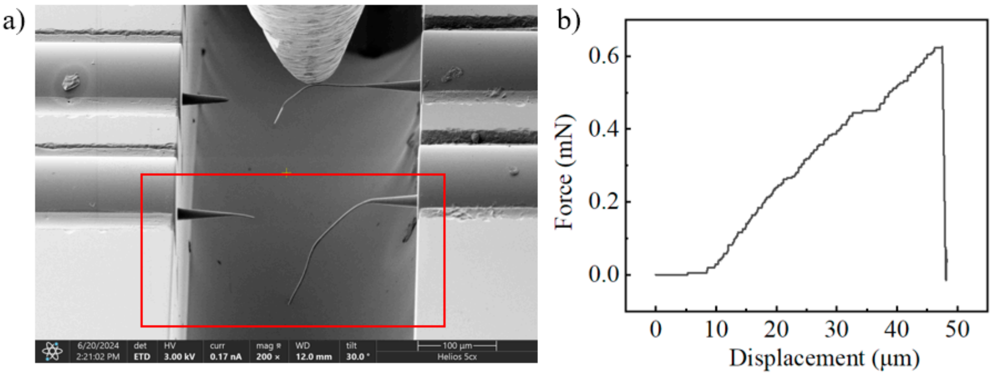

Figure S10. (a) SEM images of the PWB during compression with a curvature radius of 30 μm. (b) Force-displacement curves of the PWB under experimental conditions.

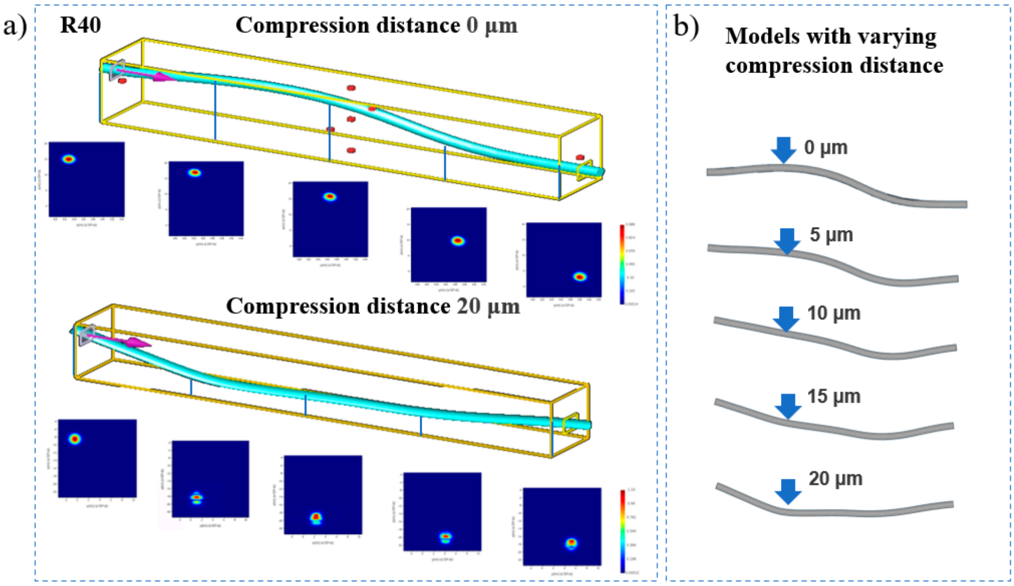

Figure S11. The simulation values of the optical loss of photonic wire bonding with varying curvatures under compression (a). Models with varying compression distance (b).

Disclaimer/Publisher's Note: The statements, opinions and data contained in all publications are solely those of the individual author(s) and contributor(s) and not of MDPI and/or the editor(s). MDPI and/or the editor(s) disclaim responsibility for any injury to people or property resulting from any ideas, methods, instructions or products referred to in the content.
